# Feasibility, Adherence, Acceptance and Usability of a Multimodal Telemonitoring for Pediatric Post-COVID Syndrome: A Bicentric Pilot Study

**DOI:** 10.1007/s10916-026-02409-x

**Published:** 2026-05-09

**Authors:** Zoe S. Oftring, Jeremy Schmidt, Julia Greenfield, Matthias Hägele, Armin Farzaneh, Eckard Hamelmann, Uta Behrends, Sebastian Kuhn

**Affiliations:** 1 Institute for Digital Medicine, Philipps University Marburg and University Clinic Giessen & Marburg, Marburg, 35042 Germany; 2 Department of Pediatrics, University Hospital OWL, Evangelisches Klinikum Bethel, University Bielefeld, Bielefeld, Germany; 3 Department of Pediatrics, University Clinic Giessen & Marburg, Marburg, Germany; 4 Children’s Hospital, TUM School of Medicine, MRI Chronic Fatigue Center for Young People (MCFC), Technical University of Munich and Munich Municipal Hospital Schwabing, Munich, Germany

**Keywords:** Telemonitoring, MHealth, Pediatrics, Post-COVID Syndrome, Digital Medicine, Wearables

## Abstract

**Abstract:**

Existing healthcare infrastructure struggles to meet the complex care required for pediatric Post-COVID Syndrome (pPCS). Telemonitoring offers potential to enhance care access, reduce patient burden, and ensure continuity. This study introduces and evaluates a novel, multimodal telemonitoring concept for pPCS with high translational potential for broader pediatric chronic and post-infectious conditions. Telemonitoring included a patient app, digital sensors (spirometer, smartwatch), Patient Reported Outcome Measures, chat/video consultations (VC), and a medical telemonitoring platform. Patients aged 12–17 years with diagnosed PCS were recruited from two pPCS outpatient university clinics in Bielefeld and Munich, Germany. Monitoring lasted three months. Evaluation focused on feasibility, adherence, acceptance, and usability, using monitoring data, the System Usability Scale (SUS), Technology Usage Inventory (TUI), and a custom survey completed by patients and parents. 30 patients (mean age: 15y ± 1.9; 57% female (17/30); mean Baseline Bell-Score: 36.4) and 30 parents participated. Adherence was high, with an average of 3.4 (smartwatch) to 4.6 (spirometry) measurements/week. Questionnaire response rate was 86% (411/480) and 97% (58/60) of VCs were conducted. SUS scores indicated very high usability (patients: 81.25/100; parents: 75.42/100). TUI results showed low skepticism, and high interest. Telemonitoring supported symptom management independent of in-person visits, despite sensor connectivity issues. This is the first study to demonstrate successful integration of telemonitoring in pPCS, with high adherence and positive feedback from all stakeholders supporting its potential. Despite occasional technical challenges and resource needs, this concept shows promise for broader hybrid telemonitoring care implementation in PCS and other post-infectious syndromes.

**Trial Registration:**

German Clinical Trials Register (DRKS), trial registration number: DRKS00029354. Registered 07 February 2023 - Retrospectively registered https://drks.de/search/en/trial/DRKS00029354/entails.

**Supplementary Information:**

The online version contains supplementary material available at 10.1007/s10916-026-02409-x.

## Background

Following acute SARS-CoV-2 infection, 0.8%–13.0% of children and adolescents develop Post-COVID Syndrome (PCS) [1–3]. According to the National Institute for Health and Care Excellence (NICE) and the World Health Organization (WHO), PCS is defined as new or ongoing symptoms three months post SARS-CoV-2 infection, lasting at least two months without other causes [4, 5]. PCS involves fluctuating, multisystem symptoms [6, 7], mainly pulmonary or cardiac complaints, fatigue, neurocognitive problems (e.g., brain fog), dizziness, sleep problems, and pain [8, 9]. A subgroup experiences Post-Exertional Malaise (PEM) [10, 11], a disproportionate symptom worsening after physical, mental, or emotional stress, risking long-term deterioration or “crashes” [12–14]. PEM is the cardinal symptom of Myalgic Encephalomyelitis/Chronic Fatigue Syndrome (ME/CFS) [15, 16], which can develop from PCS and other Post-Acute Infection Syndromes (PAIS) [17]. Autonomic dysregulation, especially Postural Orthostatic Tachycardia Syndrome (POTS) [18–20] and Mast Cell Activation Syndrome [21] are common. Psychological distress often develops secondarily due to severe daily limitations.

Despite advancing research, PCS pathophysiology remains unresolved. Current research focuses on various pathologies including autoimmunity, persistent inflammation, endothelial damage/microcirculatory dysfunction, and mitochondrial dysfunction, with different clusters seeming likely [22–24]. With absent biomarkers, diagnosis relies on clinical guidelines, symptom questionnaires, laboratory and (exclusion) diagnostics [17]. Pulse and blood pressure measurement (e.g., tilt-table test) support POTS diagnosis; heart rate variability (HRV), as index of neurocardiac function, may serve as a proxy for fatigue severity in ME/CFS and PCS [25–28]. Treatment is symptom-based, combining pharmacologic and non-pharmacologic approaches (e.g., analgesics, inhalants, circulatory drugs, antihistamines, physiotherapy [3, 22, 29]). For PEM, *pacing* supports minimizing crashes and further deterioration [30]. Further therapies are under investigation in adults [31–33].

PCS demands complex, multimodal care in specialized centers [14, 34, 35]. However, insufficient knowledge in healthcare professionals delays appropriate treatment and risks symptom deterioration through misguided recommendations (e.g., exercise training causing crashes). In Germany, few specialist outpatient clinics exist [36], resulting in long waiting times, distant travel, and insufficient long-term care.

Telemonitoring has the potential to bridge this care gap via hybrid, decentralized care. It enables real-time, longitudinal symptom tracking [37], timely physician interaction, and individualized treatment adjustments, particularly valuable in fluctuating conditions (PEM, POTS). It may inform digital biomarkers or disease trajectory research, and connect patients to distant expert centers [38]. For severely affected, house- or bed-bound patients, distant travel risks crashes with further deterioration, leaving home visits and telemedicine as the only ethically justifiable care option.

The COVID-19 pandemic catalyzed telehealth adoption in adult [39–41] and pediatric medicine [42, 43], streamlining regulations and reimbursement. Telemonitoring is now integrated into adult chronic care (e.g., diabetes, chronic heart failure) [44–46] but remains underutilized in pediatrics [47]. Prior studies primarily focused on pediatric pulmonary [48–50], cardiac [51], and mental conditions [22].

Several features distinguish pediatric from adult telemonitoring [52]: it targets both adolescents *and* caregivers, who often display “everyday digital competence” but may lack “professional competence” with digital health technologies [53], necessitating targeted education [52]. Caregivers usually operate the tools rather than patients, raising legal and ethical issues. Tools must be age-appropriate in design and functionality, ensure accurate measurements (e.g., sensor sizes, reference values, child safety), and eventually promote the gradual handover of care responsibility to adolescents.

To date, no studies have explored telemonitoring in pediatric PCS (pPCS) or ME/CFS, aside from one on acute COVID-19 [54] and another using phone follow-ups for Post-COVID sequelae [55]. Despite the potential of telemonitoring and the clinical need to acquire long-term data for PCS, no research on telemonitoring for pPCS or ME/CFS exists thus far.

This study addresses the gap by introducing and evaluating a novel, hybrid app-based telemonitoring concept integrated into routine care at two German university pPCS outpatient clinics. We present results on feasibility, adherence, digital monitoring data, patient- and caregiver-reported outcomes on overall experience, acceptance and usability.

## Methods

### Aim, Design and Setting

This exploratory bicentric pilot study, *coverCHILD Telemonitoring*, was conducted at two pediatric university hospitals in Germany to investigate the integration of a telemonitoring concept [56] as a complementary therapy component into the existing PCS outpatient services. Telemonitoring spanned October 2022–July 2023 (Bielefeld, site 1) and January 2023–August 2023 (Munich, site 2).

The primary aim was to assess the feasibility of app-supported telemonitoring in 30 children and adolescents with PCS by analyzing adherence, acceptance, user behavior, promoters and barriers, impact on care, usefulness, user-friendliness, and ease of integration into daily life from patients’ and parents’ perspectives. Secondary outcomes involved descriptive symptoms and health parameter analysis.

Study teams met weekly to exchange implementation experiences; technical coordination with the app developer occurred biweekly.

## Recruitment

Patients attending the PCS outpatient clinics received a comprehensive multidisciplinary assessment (pediatrician, pediatric psychotherapist, nurses) consisting of anamnesis, and guideline-compliant diagnostic and laboratory tests [3]. Patients fulfilling the eligibility criteria (age 12–17 years, PCS diagnosis per WHO criteria, German-speaking, smartphone ownership) were invited to participate in telemonitoring. Written informed consent was obtained from parents and patients.

## App-based Telemonitoring System Architecture

**Fig. 1:**
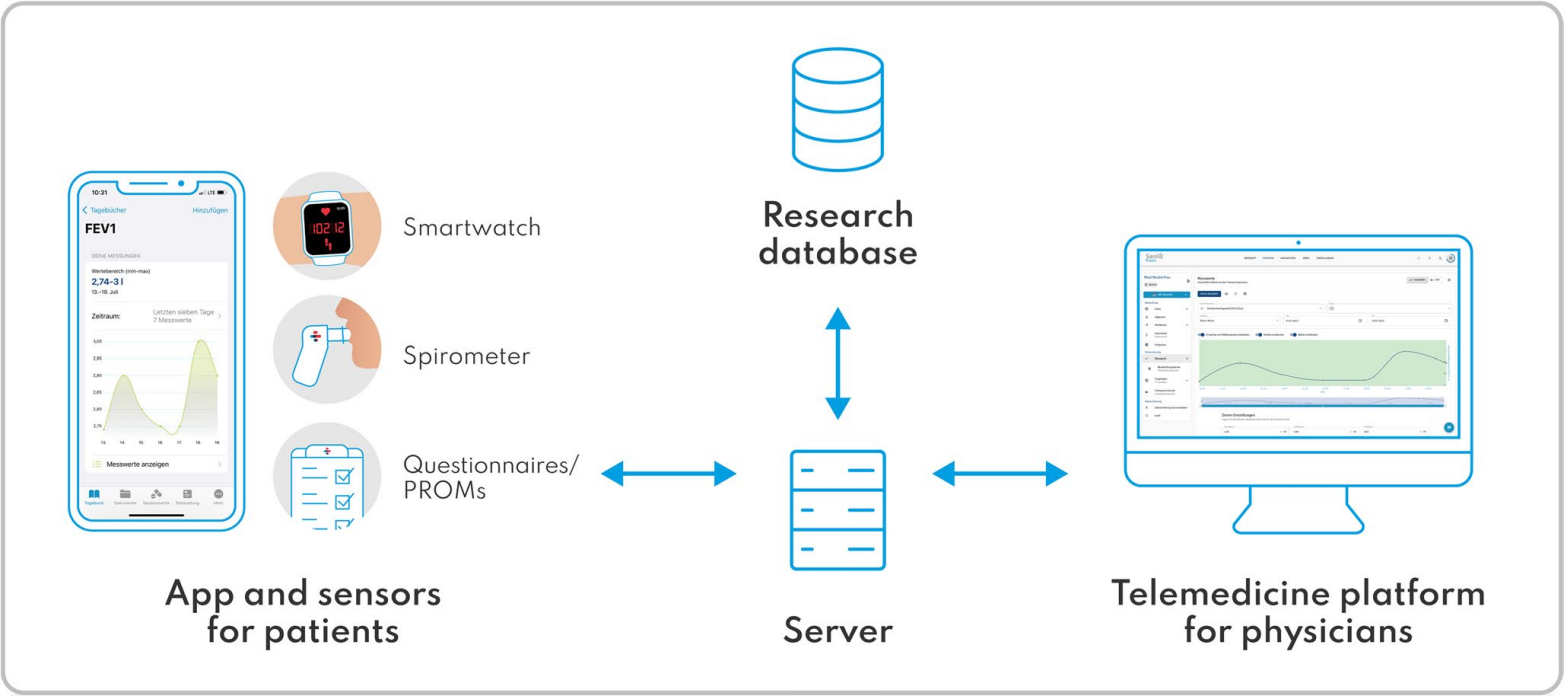
Schematic overview of the telemonitoring framework, illustrating the integration of patient-facing components (mobile app and sensors), secure data exchange and export, and the telemedicine platform for physicians

Telemonitoring comprised different layers relating to data acquisition, mobile client usage, backend application, and clinician presentation. It consisted of the patient app SaniQ (Qurasoft GmbH, Koblenz, Germany), a certified class I medical device according to the Medical Devices Directive, which was paired with digital sensors (Table 1), and a web-based portal for physicians (SaniQ telemedicine platform) (see Fig. 1). App functions included vital parameter measurement, Patient Reported Outcome Measures (PROMs) [57–60] (Table 1), data exchange and export, chat and video consultation (VC). The backend components included the server-side infrastructure that was responsible for secure data transfer, processing, storage, and visualization within the telemonitoring platform. A detailed overview of the system’s architecture is provided in Appendix 1.

During the onboarding process in the outpatient clinic, patients downloaded the app to their smartphone from the Apple App Store (iOS) or Google Play Store (Android). They created an individual user account and connected it to the telemonitoring platform. This involved a two-step approach, using an QR code provided by the physicians, together with SMS-based two-factor authentication. Two sensors were then paired to the patient’s smartphone via Bluetooth for non-invasive vital parameter measurement. Lung function (spirometry) was assessed once daily through peak expiratory flow (PEF) and forced expiratory volume (FEV1) using the MIR Smart One Oxi portable spirometer (MIR Smart One, Rome, Italy). Step count, Heart Rate (HR) and Heart Rate Variability (HRV) were ambiently measured with the Garmin Venu Sq smartwatch (Garmin Ltd, Olathe, KA, USA). Patients were requested to wear the watch throughout the day, removing only for activities in which it could be damaged, and for sleep. The app collected smartwatch and lung function data directly from the devices using the Garmin Health and MIR Application Programming Interfaces (APIs) and standard Software Development Kits (SDKs). This facilitated immediate data retrieval from the wearable, avoided data transmission to the manufacturer’s cloud infrastructure, and consequently ensured data protection compliance. Data synchronization between the sensors, the app, and the telemedicine platform was conducted automatically.

Asynchronous chat allowed communication between patients and physicians. Physician checked in at least biweekly on medical and technical issues. VCs occurred every four weeks via the app and online telemonitoring platform, reviewed health status and addressed questions. Patients participated alone or with parents. Additionally, patients were asked to perform a 1-Minute Sit-to-Stand Test (S2S) [61] with pre- and post-lung function measurements under medical observation during the VC. Participation in the test was voluntary and could be declined depending on current symptom severity.

All data (measurements, questionnaire responses, documents, chat) were transmitted in near real-time through a General Data Protection Regulation (GDPR)-compliant interface to the Qurasoft telemonitoring platform, which is hosted on encrypted servers located in Germany (see Fig. 1 and Appendix 1). For data review, physicians logged in to the telemonitoring platform using their individual username and password.

**Table 1:** A summary of the digital sensors and PROMs used for disease monitoring during the 12-week telemonitoring period

Measurement type	Device/Questionnaire	Measured parameter	Frequency
Digital sensor	Lung function: MIR Smart One Oxi spirometer^a^	Forced expiratory volume (FEV1)	Once daily
		Peak expiratory flow (PEF)	
	Activity: Garmin Venu Sq smartwatch^b^	Heart rate (HR)	Constant daily background monitoring*
		Heart rate variability (HRV)	
		Step count	
PROM	Fatigue Severity Scale (FSS) [58]	Assessment of fatigue severity	At weeks 0, 4, 8 and 12
	DePaul Symptom Questionnaire-Post-Exertional Malaise (DSQ-PEM) [59]	Symptom assessment specific to patients with PEM	
	Bell-Score [60]	Scale of fatigue and disability in CFS patients	
	Custom-made questionnaire for school attendance^c^	School attendance over the last 4 weeks	

PROM: Patient Reported Outcome Measurement, PEM: Post-Exertional Malaise, CFS: Chronic Fatigue Syndrome.^a^ MIR - Medical International Research S.P.A., Rome, Italy. ^b^ Garmin Ltd, Olathe, KS, USA. *Patients were requested to wear the watch throughout the day, removing it only when necessary. ^c^ added at study week 8 (for questionnaire see Appendix 2).

**Fig. 2:**
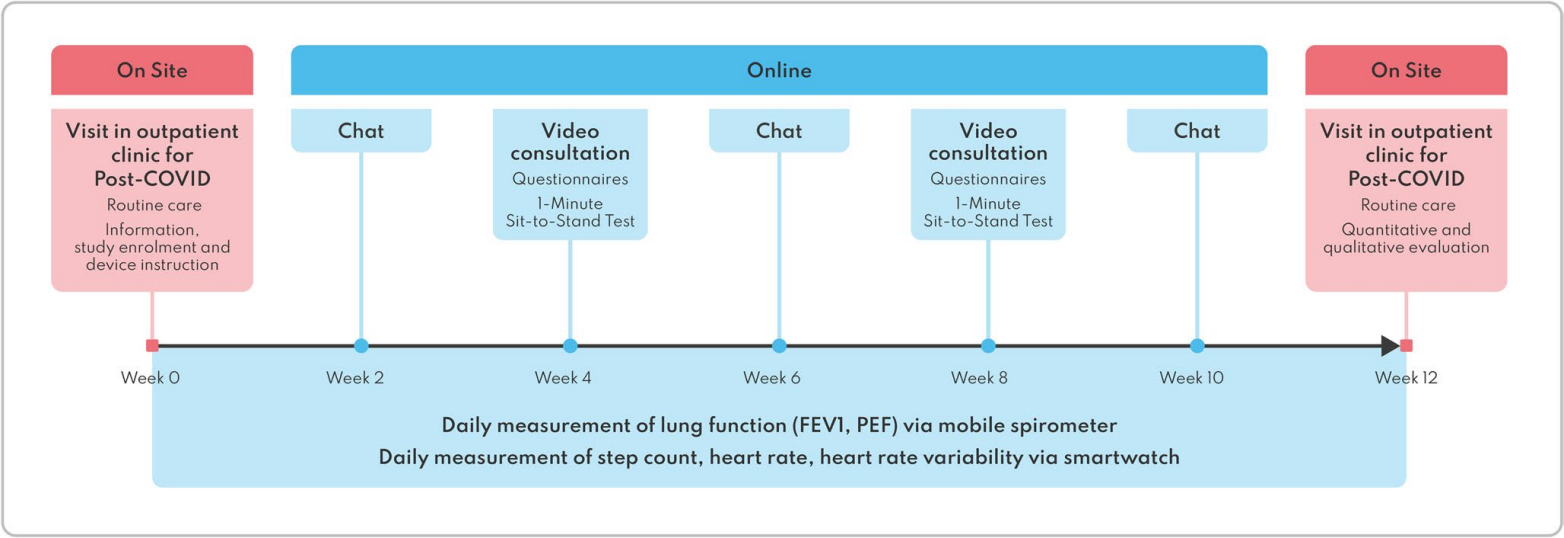
Flowchart of the three-months telemonitoring study concept, illustrating the hybrid care pathway combining on-site visits with remote vital parameter monitoring, PROMs and remote patient–physician interactions

Monitoring spanned three months ending with an on-site follow-up (see Fig. 2) or in severely affected patients as video consultation. Selection of the app, integrated functions, parameters, PROMs and questionnaires were based on current scientific knowledge about and medical guidelines for pPCS and ME/CFS.

## Evaluation

We evaluated the telemonitoring intervention by adopting a mixed-methods approach. Quantitative evaluation consisted of telemonitoring health data analysis and questionnaires (outlined below). All questionnaires, except the SUS, were adapted linguistically to create separate patient and parent versions who completed the evaluation independently to avoid influence. Qualitative evaluation was based on semi-structured interviews (results will be published separately).

## Quantitative Evaluation

### Health Data & Adherence

Lung function data was available as one value per parameter (PEF, FEV1) per day, step count data as step sum per day, heart rate data as value per minute and for analyses averaged to obtain mean daily HR. Missing values were not imputed. Adherence was analyzed through measurement frequency, questionnaire response, and chat/VCs usage. We defined ≥3 measurements/week as adherent.

### User Experience

User experience was evaluated via three instruments:

#### Technology Usage Inventory (TUI) [62, 63]

Of the 33 items (7-point Likert scale; 1 = does not apply – 7 = does apply), 20 were selected from five relevant categories (Curiosity, Anxiety, Interest, Skepticism, Accessibility) including one Utility item. Overlapping or irrelevant categories (Usefulness, Immersion, Intention to Use) were excluded. Since the validated TUI exists only in German, the authors have translated it into English for publication purposes.

#### System Usability Scale (SUS) [64]

This 10-item tool was used to assess usability (5-point Likert scale; 1 = strongly disagree – 5 = strongly agree). If parents never used the app with their child, they were asked to skip this questionnaire.

#### Custom questionnaire

To assess aspects specific to (Post-) COVID-19 telemonitoring, our team developed this questionnaire during the prior *COVID-19@home* project [65], adapting it for the pPCS context. It covers six dimensions (monitoring autonomy, application/implementation, daily integration, expert contact, overall rating, recommendation readiness) using binary (yes/no) and Likert scales. Participants also rated their own or their child’s usage independence (11-Item-Likert scale; 0 = always with support – 10 = always alone) and provided socio-demographic data. The original German version has not yet been validated in English (see Appendix 3,4).

## Data and Statistical Analysis

All analyses were performed descriptively. Primary endpoints included number of days with documented lung function and smartwatch data, VCs, S2S, and completed questionnaires. PROMs were calculated according to their evaluation matrix. Sensor data and symptom questionnaire scores were examined for changes over the study period to identify trends. Subgroup analyses (ages 12–14 vs. 15–17) regarding measurement adherence were performed. TUI scores were summed per category (range: 3–21 or 4–28, depending on category), with means and standard deviations (SD) reported. SUS scores were transformed to a percentile score between 0 and 100 (≥ 68 indicates usability = grade C; ≥ 80.3 indicates excellent usability = grade A) [66]. Custom questionnaire responses were reported descriptively.

## Results

### Study Population

Overall, 30 patients participated in the telemonitoring study (center 1: 20, center 2: 10), all of whom completed the 12-week monitoring period without dropouts. Participants were predominantly adolescents (mean age 15.0 ± 1.9 years), with 57% female (17/30), and showed moderate to severe illness (mean Bell-Score 36.4), high fatigue (mean FSS 6.1), and high PEM prevalence (87%; 26/30). The Canadian Consensus Criteria for ME/CFS [67] were met by 40% (12/30), and POTS was prevalent in 63% (19/30). Further sociodemographic and clinical baseline characteristics are detailed in Table 2 (patients) and Appendix 5 (parents).

**Table 2:** Sociodemographic and clinical characteristics of patients at baseline (*n* = 28; *missing = 2)

Patient characteristics			Value
Sociodemographic characteristics			
	Age in years, mean ± SD		15.0 ± 1.9
	Gender, female, n (%)		17 (57)
	Nationality, n (%)*		
		German	27 (90)
		Other	1 (3)
	School type*, n (%)		
		Higher education	26 (93)
		Vocational school/apprenticeship	2 (7)
Clinical characteristics at inclusion			
	Presence of allergies, n (%)		13 (43)
	Presence of SARS-CoV-2-Vaccination prior infection, n (%)		19 (63)
	Hospital treatment during acute COVID-infection, n (%)		0 (0)
	Presence of pathological lung function at baseline, n (%)		5 (17)
	Time between COVID-19 infection and study inclusion, months, mean ± SD		10.0 ± 5.68
	Presence of PEM^a^, n (%)		26 (87)
	Presence of ME/CFS^b^, n (%)		12 (40)
	Presence of POTS^c^, n (%)		19 (63)
	Fatigue Severity Scale, mean ± SD		6.1 ± 1.1
	Bell-Score, mean ± SD		36.4 ± 14.5
	School absence due to PCS 4 weeks^d^ prior to study inclusion in days, mean ± SD		9.8 ± 8.1

^a^ based on DSQ-PEM [59].

^b^ based on the Canadian Consensus Criteria [67]. ^c^based on a 10-minute standing test [68,69, pp111-112] in connection with symptoms and medical assessment. ^d^ result interpretation should consider that four school weeks correspond to 20 school days (4 × 5 days), meaning that patients missed 9.8/20 (49%) school days on average.

### Telemonitoring Adherence

Spirometry data were reliably transferred between the sensors, app and telemedicine platform, and recorded on average on 4.6 days per week (66%) (Table 3).

Regarding smartwatch data, patients transmitted on average 3.4 days of pulse data/week (49%) and 3.3 days for step data/week (47%). Smartwatch data transmission proved more complex than expected. Despite proper use by patients, technical problems impeded data transmission to the telemedicine platform. HRV data was only insufficiently recorded due to large volumes of data that could not be supported by Bluetooth transfer at the time, and hence excluded from monitoring and evaluation.

**Table 3:** Patient adherence to telemonitoring during the 12-week-monitoring period (*n* = 30)

Monitoring data		Value
Adherence to telemonitoring component, n (%)		
	Both video consultations conducted	58/60 (97)
	Chat function utilized at least once	30/30 (100)
	Completed symptom questionnaires^a^, total	411/480 (86)
	DSQ-PEM	110/120 (92)
	FSS	110/120 (92)
	Bell-Score	104/120 (87)
Data transfer, days per week per patient, mean ± SD		
	Transmitted spirometry data, global, weeks 1–12	4.6 ± 1.34
	Transmitted spirometry data, weeks 1–4	5.6 ± 1.48
	Transmitted spirometry data, weeks 5–8	4.8 ± 1.98
	Transmitted spirometry data, weeks 9–12	3.4 ± 1.91
	Transmitted spirometry data, days in total^b^, n (%)	55/84 (66)
	Transmitted pulse data, global, weeks 1–12	3.4 ± 2.17
	Transmitted pulse data, weeks 1–4	4.1 ± 2.52
	Transmitted pulse data, weeks 5–8	2.9 ± 2.55
	Transmitted pulse data, weeks 9–12	3.1 ± 2.51
	Transmitted pulse data, days in total^b^, n (%)	40/84 (48)
	Transmitted steps data, global, weeks 1–12	3.3 ± 2.09
	Transmitted steps data, weeks 1–4	4.0 ± 2.43
	Transmitted steps data, weeks 5–8	2.9 ± 2.41
	Transmitted steps data, weeks 9–12	3.0 ± 2.40
	Transmitted steps data, days in total^b^, n (%)	40/84 (48)

^a^ Based on the four listed questionnaires below at four measuring points (week 1 study inclusion, week 4 and 8 video consultation, week 12 end of study). ^b^ In reference to a total of 84 study days for one patient during the 12-week study period.

As spirometry provided the most reliable data, it was used for a time- and age-based subgroup analysis (12–14 vs. 15–17). Overall, measurement frequency declined over time: from 5.6 measurement days/week (month 1) to 3.4 (month 3). Smartwatch data saw similar trends but was less reliable due to the mentioned technical issues.

**Fig. 3:**
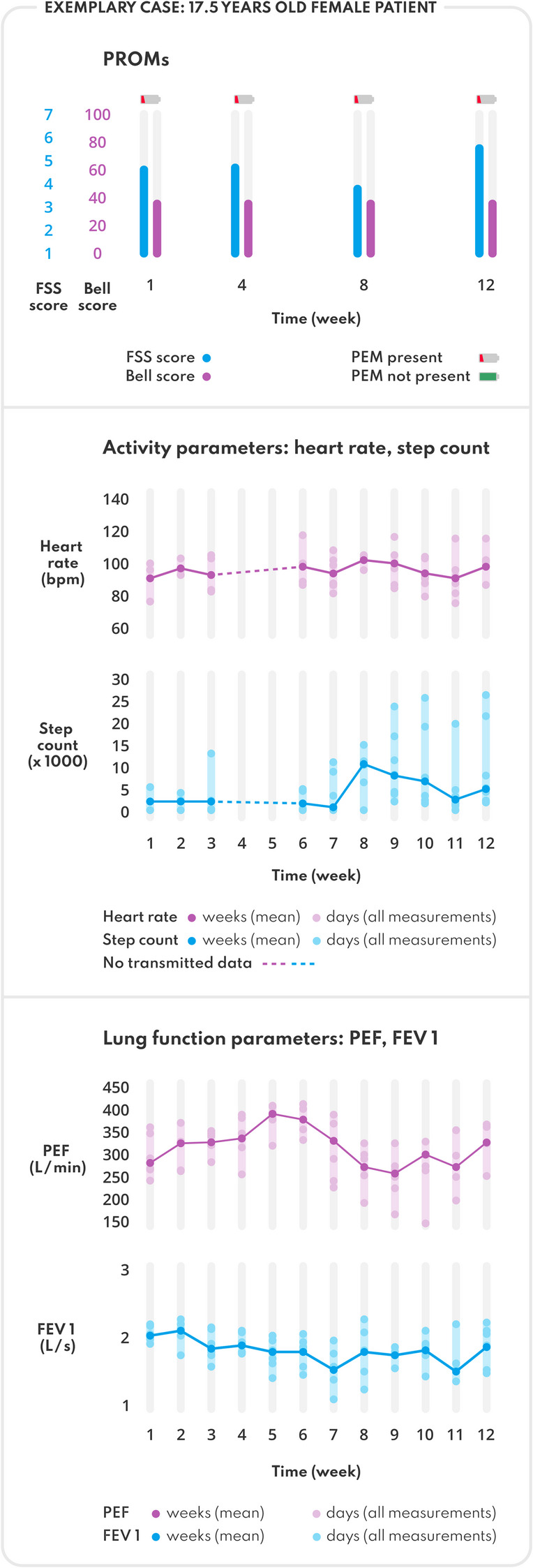
Exemplary 12-week telemonitoring trajectory from a representative adolescent patient with Post-COVID Syndrome, illustrating longitudinal trends in activity parameters, respiratory parameters, and patient-reported outcome measurements. Data include the Fatigue Severity Score (FSS), the presence of Post-Exertional Malaise (DSQ-PEM) and the Bell Score, along with heart rate, step count, peak expiratory flow (PEF), and forced expiratory volume (FEV1)

Subgroup analysis showed little difference (month 1: 5.8/week (younger) vs. 5.4/week (older); month 3: 3.3/week (younger) vs. 3.7/week (older). Figure 3 illustrates the 12-week digital data trajectory in one representative patient.

### Questionnaires and PROMs

Patients were asked to complete all four PROMs at four time points, totaling in 480 possible responses. Overall, completion was 86% (411/480), with highest rates for FSS and DSQ-PEM (92%; 110/120), followed by Bell-Score (87%; 104/120) and school attendance (73%; 87/120). The latter was added 8 weeks after study start, impacting response rates. Integration into the app was seamless; in-app reminders worked well, with occasional additional prompts via chat.

### Communication

All patients used the chat successfully, with frequency ranging from minimal (VC appointment coordination) to frequent medical and technical inquiries. Parental use was rare and only present in younger patients. Physicians typically responded within one week. Each patient was scheduled for two VCs, with 97% (58/60) completed despite occasional scheduling issues, mainly due to short-notice changes (patients) and shift work (doctors). Missed appointments were due to patients not attending or failure to schedule despite reminders. VCs lasted 20–60 min and addressed health status, therapeutic readjustments, and telemonitoring feedback. In 76% (44/58) of VCs, patients performed a 1-min Sit-to-Stand test with pre/post spirometry. No adverse events occurred. Participation was optional and declined (24%) when patients felt too exhausted. Technical interruptions were rare and managed via app audio or phone consultation. The spirometry test also served to review spirometry technique and offer feedback.

### Evaluation of the Telemonitoring Experience

All three evaluation questionnaires were completed by 28/30 patients and 28/30 parents.

#### Technology Usage Inventory

**Fig. 4:**
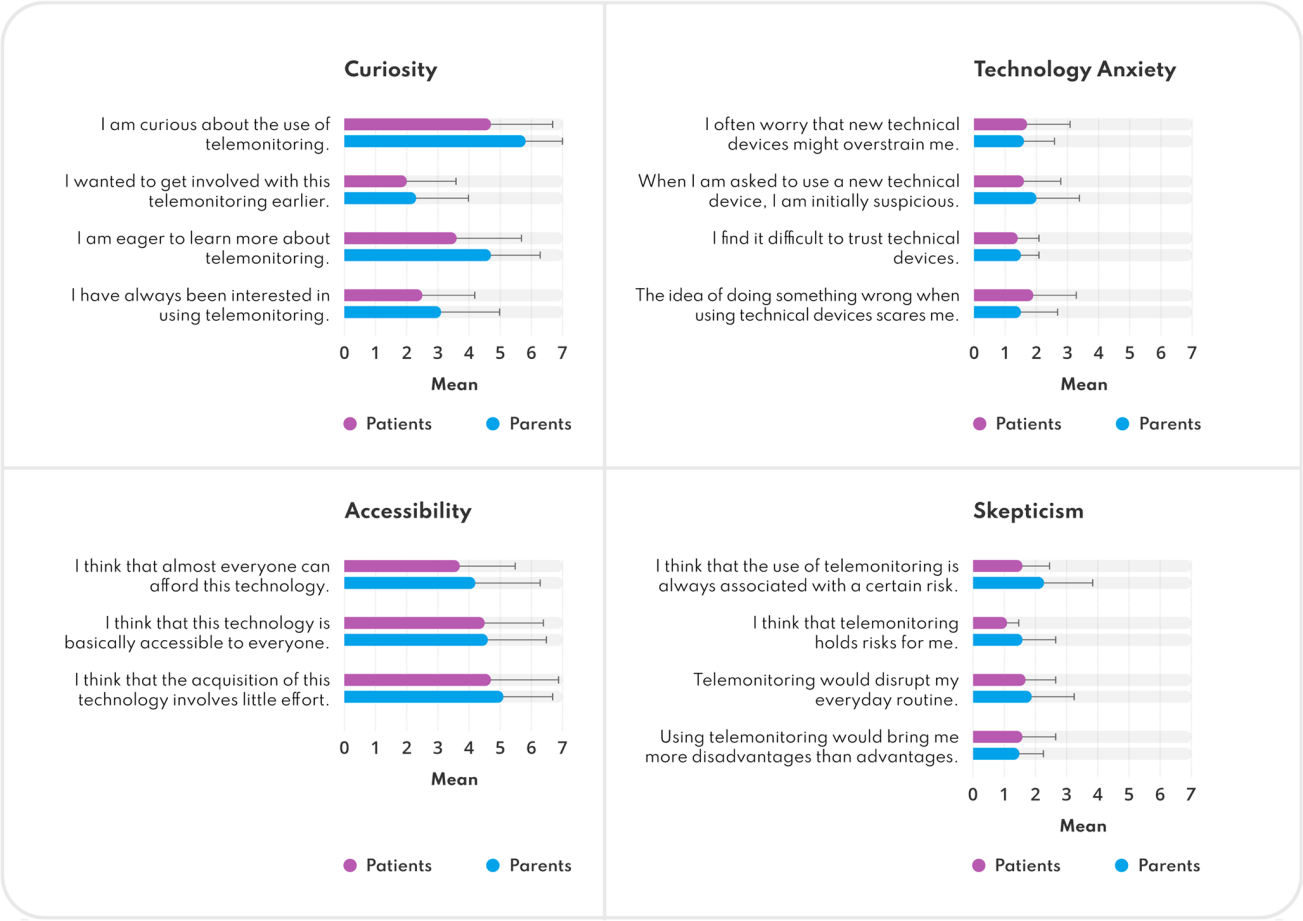
Results from the Technology Usage Inventory, divided into four categories and their associated items. Results are depicted as mean value (bar) and standard deviation (error bars) per item as rated by patients (*n* = 28; purple) and parents (*n* = 28; blue)

Patients and parents reported low Technology Anxiety and Skepticism, and high Curiosity, Interest, and Accessibility (Table 4). Parents noted minimal acquisition effort. Both groups agreed on the perceived utility and would consider purchasing it. Ratings were generally consistent (see Fig. 4 and Appendix 6).

**Table 4:** Results of the Technology Usage Inventory, patients (*n* = 28) and parents (*n* = 28) evaluation for the polled categories

TUI Category^a^	Max. poss. Sum Score	Sum Score^b^, mean ± SD		Range, min–max		Missing, *n*	
		Patients	Parents	Patients	Parents	Patients	Parents
Curiosity	28	12.6 ± 6.2	15.7 ± 4.0	4–28	9–24	3	3
Technology Anxiety	28	6.6 ± 3.8	6.7 ± 2.8	4–19	4–16	2	2
Interest	28	13.0 ± 6.4	13.0 ± 5.4	1^c^–25	4–25	3	3
Skepticism	28	5.8 ± 2.6	7.21 ± 3.4	4–14	4–15	3	2
Accessibility	21	12.5 ± 4.9	13.9 ± 4.3	3–21	6–21	3	2

^a^As this questionnaire polled only one item from the category Utility, no sum score was calculated, and this category is thus not listed here. The results are mentioned in the paragraph above. ^b^ Higher sum scores indicate strong representation of the corresponding aspect among respondents. Conversely, low values mean that the aspect is less relevant among respondents. ^c^ One patient answered only one item in the Interest category leading to a minimum sum score of 1. Max. poss.= Maximal possible.

#### System Usability Scale

Patients rated the telemonitoring’s usability at a mean SUS score of 81.25 (SD ± 15.67), corresponding to grade A. Of 28 parents, 17 (61%) had used the system and rated it at 75.42 (SD ± 22.17), corresponding to grade B.

#### Custom Questionnaire

Telemonitoring was well received. Both patients and parents reported smooth daily integration, minimal uncertainty despite little experience with previous digital health monitoring, and high levels of independent use among children.

**Fig. 5:**
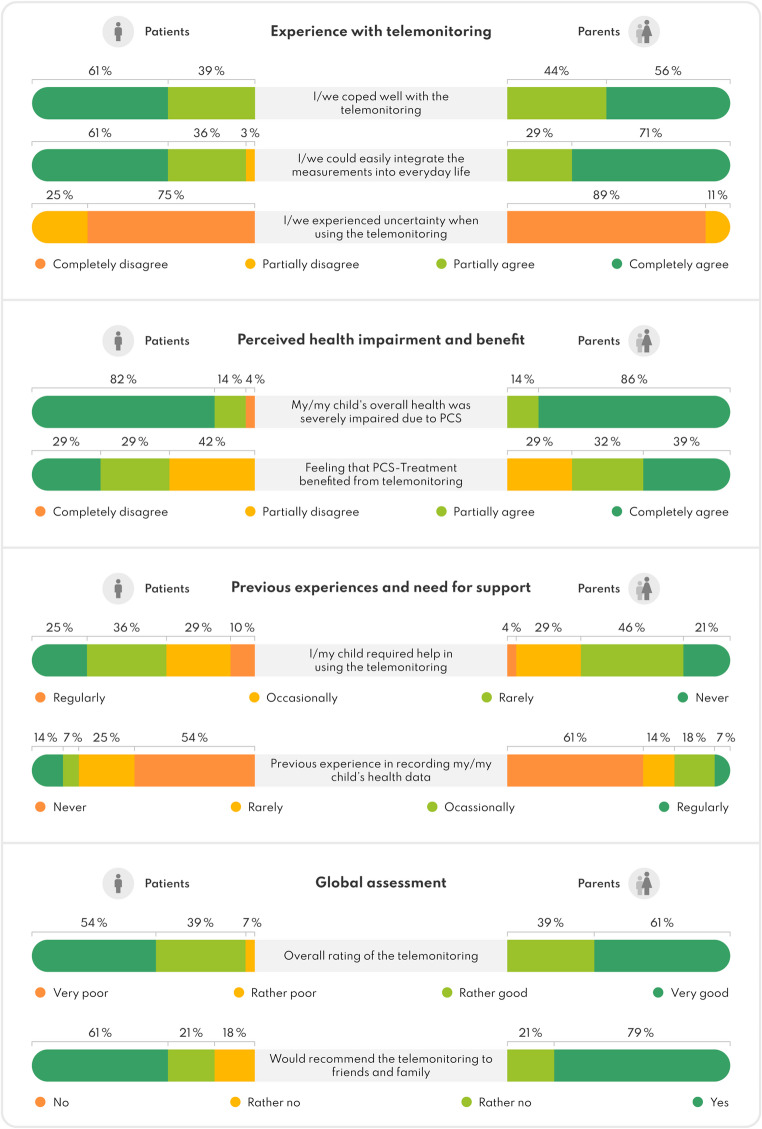
Graphic illustration of patients’ (*n* = 28; left) and parents’ (*n* = 28; right) experience with the telemonitoring and overall assessment based on results from our custom questionnaire. Percentages have been rounded

Therapeutic benefit was completely or partially perceived by 57% (16/28) of patients and 71% (20/28) of parents. Overall ratings were predominantly ‘very good’ (patients: 54%; 15/28 vs. parents: 61%; 17/28) or ‘good’ (both 39%; 11/28). Only two patients rated it ‘rather poor’. Most would recommend the telemonitoring, with slightly higher endorsement among parents (see Fig. 5).

## Discussion

### Principal Results: Feasibility, Adherence, Acceptance and Usability

To the best of our knowledge, this study presents the first telemonitoring care concept for pPCS and ME/CFS. High adherence rates confirmed feasibility across all components. Spirometry was used consistently and provided detailed insights. Smartwatch adherence was slightly lower, mainly due to technical issues.

Questionnaire completion was excellent, though the Bell-Score and school attendance questionnaire saw slightly lower response rates. Patients found the Bell-Score inconvenient due to the need to open it in a separate app section, and some may have considered the school questionnaire irrelevant as their illness prevented them from attending school completely, underscoring the need for tools that patients find useful to their care and a straightforward, user-friendly interface, especially given neurological symptoms.

Patients reliably engaged with chat messages and VCs, and remote functional testing (Sit-to-Stand) was successfully performed. Our study therefore both supports prior evidence of remote assessments in pediatrics [42] and extends it by demonstrating safe performance of the Sit-to-Stand test even in a vulnerable population. The voluntary nature of such tests remains essential especially considering the presence of PEM. Parental supervision emerged as a facilitator, enhancing safety, improving performance, and reducing explanation time, especially for younger children.

A key strength of our study is the use of medically certified, GDPR-compliant communication tools. Earlier studies on telecommunication were limited by privacy concerns (e.g., non-GDPR-compliant platforms like Zoom) [42] or less sophisticated methods like phone calls [55], and the only telemonitoring study in adult PCS lacked a communication component [70].

Most patients used the telemonitoring components independently, but the need for assistance was more common in younger children (e.g., participation in VC, messaging the physician), while parents acted primarily as observers. Our results counteract concerns about feasibility, adherence, and safety, reinforcing the potential for broader implementation of telemonitoring in other chronic pediatrics conditions.

### Telemonitoring and Post-COVID Syndrome Specific Care

Families and physicians perceived telemonitoring as beneficial for pPCS care. Unlike standard care, where follow-ups are often limited by resource constraints, our approach enabled regular reevaluation, aligning with PCS guidelines [22, 71]. While 57% of patients and 71% of parents rated telemonitoring as beneficial for PCS treatment, this should be interpreted in the context of a short monitoring period compared to long healing processes, the absence of curative treatments, and its role as a care enhancement, not as treatment intervention. The strongest improvement thus lies in “continuous, closer care” compared to “little or no care at all”.

Three to four data points per week, regular PROMs and VCs offer far more detailed insights into disease trajectories than standard quarterly follow-ups, marking a significant advance in pPCS and ME/CFS care. Clinical recommendations are also starting to consider smartwatch integration to avoid PEM [72] and specific apps are evolving (examples include: Visible, WellTory, and FimoHealth). This aligns with prior findings that telemonitoring may even improve treatment adherence in pediatric patients [50, 52] and provide benefits for pediatric conditions [38, 42, 43, 47, 55]. Lastly, most participants rated the telemonitoring concept as very good or good and would recommend it. Only two patients expressed dissatisfaction, due to wristband skin irritation, and need for technical/parental support. Overall, the high acceptance echoes existing literature on the effectiveness and user-friendliness of telemedicine in pediatric chronic care [73].

### Strength and Challenges of Telemonitoring

A key strength of our telemonitoring approach was its integration of multiple monitoring components into one platform, enabling structured evaluation. While no validated digital biomarkers for PCS exist yet, physicians considered the collected data valuable “soft” digital markers for symptom tracking and therapeutic guidance.

Telemonitoring helped mitigate access barriers to PCS care arising from limited healthcare resources. We also argue that telemonitoring supported preventing PEM, which often follows exhausting in-person visits. Future studies are needed to investigate the correlation between telemonitoring and PEM prevention further.

Despite iterative troubleshooting, technical challenges due to high data volumes, synchronized once-daily, Bluetooth instability, varying smartphone settings and software inconsistencies (mainly updates) could only partially be resolved, led to data gaps and impeded the originally planned HRV-monitoring. Therefore, findings may underrepresent smartwatch usage. Future studies should prioritize thorough pre-testing, enable reliable HRV tracking as one of the most promising biomarkers for fatigue, early detection of PEM and autonomic dysfunction, and include alerts for connectivity loss.

Telemonitoring generates large datasets, becoming increasingly difficult to process and rendering patients a “big data” challenge [74]. Our platform pre-processed data into daily step counts and hourly HR averages. Future implementations may wish to consider distinguishing between resting and active HR as this would better reflect disease activity, improve the evaluation of exercise response (S2S), support observing of vital parameter trends in relation to POTS medication, and fatigue severity. Telemonitoring requires digital competencies among physicians, for interpreting data, communicating results, and effective sensor usage [75]. Although such training initiatives exist [76–79], structural integration into medical education remains limited. Finally, patient onboarding, continuous data review, scheduling VCs, and responding to chat communication required significant physician time in already time-constrained workplaces and were only feasible through funding by an academic research grant. Given that care for pediatric PCS and ME/CFS remains widely insufficient, policy-level solutions addressing workforce capacity and reimbursement are essential. If future studies further substantiate the clinical benefit of telemonitoring in PCS, reimbursable integration into standard care, similar to established telemonitoring programs for heart failure in Germany [44–46], may represent a feasible approach to strengthening workforce resources and mitigate current capacity limitations, e.g., through task delegation of aspects such as onboarding, monitoring of data synchronization, technical support, and VC scheduling to study nurses or medical assistants in future care models.

### Limitations

This pilot study was conducted at two study sites with a short observation period and a small sample size, which may limit generalizability and underrepresent certain social groups (e.g., minorities, low education levels). As this pilot study focused on feasibility and adherence, no control group was included. Consequently, conclusions regarding added benefit over standard pPCS care are limited, despite encouraging descriptive findings. Intermittent smartwatch data transmission and the inability to evaluate heart rate variability limited the full potential of wearable-based monitoring in this pilot study setting. Nevertheless, HRV remains a key parameter in pediatric PCS, and the technical challenges encountered provide important insights for refining future telemonitoring concepts. Licensing of the telemonitoring components from the age of 12 years limits the scientific significance in younger patients. Telemonitoring also risks reinforcing the digital divide, excluding those without access to or financial means for devices or stable internet. The project was only feasible through research funding, as telemonitoring for PCS or ME/CFS is currently not reimbursed in Germany. Contributing factors include low economic incentives for pediatric care [50], complex design requirements, and higher legal and ethical hurdles.

### Future Perspective

Telemonitoring is a valuable tool for managing pPCS, however, supporting severely ill patients, especially in ME/CFS care, requires further development, including extended monitoring periods considering the longer disease course, adapted PROMs, and medication tracking. Device and parameter selection should mirror specific symptom clusters (e.g., spirometry for respiratory symptoms, blood pressure and medication tracking for POTS, alongside standard HR, HRV, and PROMs). Future studies should assess smartwatch wear time to evaluate adherence and user behavior. We are already implementing these insights in two follow-up projects *Telemonitoring for Youth ME/CFS* (TYME) [80] and *Pediatric Network for Care and Clinical Research on Post-Acute Sequelae of COVID-19 (Long COVID)*,* similar Post-Acute Infection Syndromes*,* and ME/CFS* (PEDNET-LC) (AP 4.3 TELE-LC) [81].

Broader use of digital parameters may support the identification of digital biomarkers and more personalized treatment strategies. Moreover, the potential of telemonitoring data to support biomarker research and personalized treatment or exercise-management strategies remains to be further investigated in future studies. Despite telemonitoring’s considerable potential for improving pPCS and ME/CFS care, high-quality evidence on these diseases and general pediatric telemedicine remains limited. Larger studies with extended follow-up, compare groups, quality of life assessment, and cost-effectiveness analyses are needed to support its integration into standard care and pediatric reimbursement frameworks.

For patients severely affected by PEM, telemonitoring emerges as ethically essential to avoid further deterioration triggered by in-person visits by reducing the necessity of such visits through remote alternatives and prioritize in-person visits only when specifically indicated. Future guidelines should prioritize its inclusion to support safe, continuous care.

## Conclusion

Telemonitoring is a feasible, effective, and resource-efficient addition to pediatric PCS care. It provided valuable additional information about disease trajectories, supported more responsive, demand-oriented care compared to standard care, and fostered a trustful family-clinician relationship. High adherence, usability rates and patient autonomy highlight its potential to empower young individuals in managing chronic illnesses, while detailed therapeutic benefits are still to be determined in future research. Parents reported increased reassurance, and satisfaction rates were high among all groups. However, relevant hurdles including technical challenges regarding smartwatch connectivity, and high personnel resources still need to be addressed. In summary, these findings support the broader implementation of telemonitoring as a hybrid care model for pediatric PCS, ME/CFS and chronic pediatric or postinfectious conditions.

## Supplementary Information

Below is the link to the electronic supplementary material.

Supplementary Material 1

Supplementary Material 2

Supplementary Material 3

Supplementary Material 4

Supplementary Material 5

Supplementary Material 6

## Data Availability

All relevant data is included in the manuscript. Further information is available from the corresponding author on reasonable request.

## Abbreviations

EvKB: Evangelisches Klinikum Bethel (Evangelical Hospital Bethel)
FEV1: Forced expiratory volume
GDPR: General Data Protection Regulation
HR: Heart rate
HRV: Heart rate variability
MCFC: MRI Chronic Fatigue Center for Young People
ME/CFS: Myalgic Encephalomyelitis/Chronic Fatigue Syndrome
MDD: Medical Devices Directive
NICE: The National Institute for Health and Care Excellence
NUM: German National Network of University Medicine
PAIS: Post-Acute Infection Syndromes
PCS: Post-COVID Syndrome
pPCS: Pediatric Post-COVID Syndrome
PEF: Peak expiratory flow
PEM: Post-Exertional Malaise
POTS: Postural Orthostatic Tachycardia Syndrome
PROM: Patient Reported Outcome Measure
SUS: System Usability Scale
S2S: 1-Minute Sit-to-Stand test
TUI: Technology Usage Inventory
TUM: Technical University of Munich
VC: Video consultation
WHO: World Health Organization
