# Supplementary material for: Feasibility, Adherence, Acceptance and Usability of a Multimodal Telemonitoring for Pediatric Post-COVID Syndrome: A Bicentric Pilot Study
Source: J Med Syst. 2026 May 9;50(1):76. doi: 10.1007/s10916-026-02409-x (PMC13157441; doi:10.1007/s10916-026-02409-x)
Supplement: Supplementary file 1 — Supplementary Material 1 [file 10916_2026_2409_MOESM1_ESM.pdf]

Appendix 1. System architecture of the telemonitoring system used in the coverCHILD Telemonitoring study

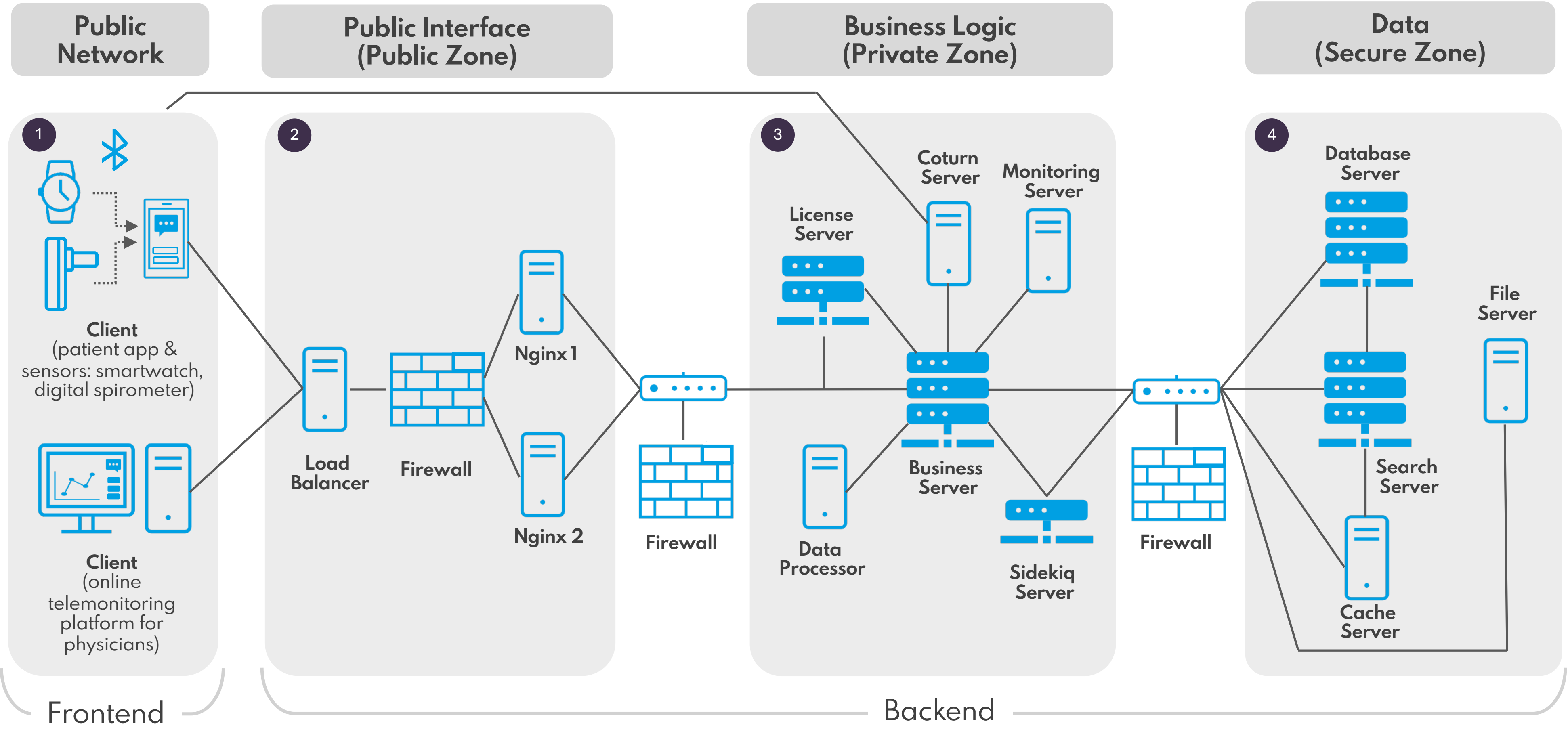

The telemonitoring system comprises different layers relating to data acquisition (smartwatch, spirometer, PROMs/questionnaires, Box 1), mobile client usage (App, telemonitoring platform, Box 1), backend application (Box 2-4), and clinician presentation and communication (vital parameters, PROMs/questionnaire responses, chat, video consultation, Box 1). In terms of data transfer and safety mechanisms within the telemonitoring system, data transfers are only permitted between neighboring zones. When crossing between zones, requests are forwarded to the corresponding router, which contains a firewall. In the public zone, the load balancer acts as an external gateway. The system architecture was provided courtesy of Qurasoft.

Graphic: Institut für Digitale Medizin, Philipps-Universität Marburg & UKGM Marburg

Institut für DIGITALE MEDIZIN Qurasoft
