# Supplementary material for: Feasibility, Adherence, Acceptance and Usability of a Multimodal Telemonitoring for Pediatric Post-COVID Syndrome: A Bicentric Pilot Study
Source: J Med Syst. 2026 May 9;50(1):76. doi: 10.1007/s10916-026-02409-x (PMC13157441; doi:10.1007/s10916-026-02409-x)
Supplement: Supplementary file 2 — Supplementary Material 2 [file 10916_2026_2409_MOESM2_ESM.pdf]

**Appendix 5.** Table presenting sociodemographic characteristics of parents (n=28; missing=2).

| **Parents' sociodemographic characteristics** | **Value, n (%)** |
|---|---|
| Living alone with the child (patient) | 5 (18) |
| Marital Status | 17 (57) |
| Married | 22 (79) |
| Relationship | 1 (4) |
| Single | 3 (11) |
| Divorced | 1 (4) |
| Not applicable (guardian of patient) | 1 (4) |
| Nationality | |
| German | 27 (90) |
| Other | 1 (3) |
| Highest Educational Degree | |
| Secondary School | 11 (39) |
| College | 5 (18) |
| University Degree | 11 (39) |
| PhD | 1 (4) |
| Employed, yes | 25 (89) |
