# Supplementary material for: Feasibility, Adherence, Acceptance and Usability of a Multimodal Telemonitoring for Pediatric Post-COVID Syndrome: A Bicentric Pilot Study
Source: J Med Syst. 2026 May 9;50(1):76. doi: 10.1007/s10916-026-02409-x (PMC13157441; doi:10.1007/s10916-026-02409-x)
Supplement: Supplementary file 3 — Supplementary Material 3 [file 10916_2026_2409_MOESM3_ESM.pdf]

Appendix 6. Table presenting patients' (n=28) and parents' (n=28) evaluation results of the Technology Usage Inventory, depicted for all individual items in each polled category.

TUI Category[a]	Score[b], mean ± SD	
	Patients	Parents
**Curiosity**		
I am curious about the use of telemonitoring.	4.7 ± 2.0	5.8 ± 1.2
I wanted to get involved with this telemonitoring earlier.	2.0 ± 1.6	2.3 ± 1.7
I am eager to learn more about telemonitoring.	3.6 ± 2.1	4.7 ± 1.6
I have always been interested in using telemonitoring.	2.5 ± 1.7	3.1 ± 1.9
**Technology Anxiety**		
I often worry that new technical devices might overstrain me.	1.7 ± 1.4	1.6 ± 1.0
When I am asked to use a new technical device, I am initially suspicious.	1.6 ± 1.2	2.0 ± 1.4
I find it difficult to trust technical devices.	1.4 ± 0.7	1.5 ± 0.6
The idea of doing something wrong when using technical devices scares me.	1.9 ± 1.4	1.5 ± 1.2
**Skepticism**		
I think that the use of telemonitoring is always associated with a certain risk.	1.6 ± 0.9	2.3 ± 1.6
I think that telemonitoring holds risks for me.	1.1 ± 0.4	1.6 ± 1.1
Telemonitoring would disrupt my everyday routine.	1.7 ± 1.0	1.9 ± 1.4
Using telemonitoring would bring me more disadvantages than advantages.	1.6 ± 1.1	1.5 ± 0.8
**Accessibility**		
I think that almost everyone can afford this technology.	3.7 ± 1.8	4.2 ± 2.1
I think that this technology is basically accessible to everyone.	4.5 ± 1.9	4.6 ± 1.9
I think that the acquisition of this technology involves little effort.	4.7 ± 1.8	5.1 ± 1.6

TUI Category[a]	Score[b], mean ± SD	
	Patients	Parents
**Interest**		
In the course of my life, I have gained a lot of technical knowledge.	4.6 ± 2.1	4.2 ± 1.6
When a new technical device comes onto the market, I inform myself about it.	2.7 ± 1.9	2.8 ± 1.6
I always try to get the latest information about new technical developments.	2.9 ± 1.7	3.1 ± 1.6
I inform myself about technological developments.	3.2 ± 1.7	3.0 ± 1.6
**Utility**		
If I could afford this technology, I would buy it.	4.9 ± 1.8	5.8 ± 1.3

[a] Each category encompasses 3 to 4 items with each item being rated on a 7-point Likert scale (1=does not apply; 7=does apply).

[b] Higher sum scores indicate strong representation of the corresponding aspect among respondents. Lower sum scores mean that the aspect is less relevant among respondents.
