# Supplementary material for: Feasibility, Adherence, Acceptance and Usability of a Multimodal Telemonitoring for Pediatric Post-COVID Syndrome: A Bicentric Pilot Study
Source: J Med Syst. 2026 May 9;50(1):76. doi: 10.1007/s10916-026-02409-x (PMC13157441; doi:10.1007/s10916-026-02409-x)
Supplement: Supplementary file 4 — Supplementary Material 4 [file 10916_2026_2409_MOESM4_ESM.pdf]

Appendix 2. School attendance questionnaire (custom).

Fragen

Frage 1
Wie viele Tage hast du in den letzten vier Wochen in der Schule gefehlt?

Frage 2
Wie viele dieser Fehltage waren nicht aufgrund des Post-Covid Syndroms, sondern hatten andere Gründe (z.B. akuter Infekt)?

Frage 3
Falls du nur eine gewisse Stundenanzahl zur Schule gegangen bist, wie viele Schulstunden (45min) warst du durchschnittlich pro Tag in der Schule?
(Optional)

Translation:

Question 1:

How many days have you missed school in the last four weeks?

Question 2:

How many of these missed days were not due to post-COVID syndrome, but had other causes (e.g. acute infection)?

Question 3:

If you only attended school for a certain number of hours, how many school hours (45 minutes) did you spend at school on average per day? [optional]
