# Supplementary material for: Feasibility, Adherence, Acceptance and Usability of a Multimodal Telemonitoring for Pediatric Post-COVID Syndrome: A Bicentric Pilot Study
Source: J Med Syst. 2026 May 9;50(1):76. doi: 10.1007/s10916-026-02409-x (PMC13157441; doi:10.1007/s10916-026-02409-x)
Supplement: Supplementary file 5 — Supplementary Material 5 [file 10916_2026_2409_MOESM5_ESM.pdf]

Appendix 3. Custom evaluation questionnaire polling patients' overall experience with telemonitoring.

ID Studien-TN: _______________ Ausfülldatum: _______________

Evaluation coverCHILD Telemonitoring

Fragebogen für Kinder und Jugendliche

Du hast in den letzten drei Monaten gemeinsam mit deinen Eltern das Telemonitoring mit App und Sensoren im Rahmen deiner Post-Covid-Erkrankung im Rahmen der Studie „coverCHILD Telemonitoring" durchgeführt. Wir würden anhand verschiedener Fragen in diesem Fragebogen gerne mehr über deine Erfahrungen mit dem Monitoring herausfinden.

Bei allen Fragen bezieht sich der Begriff „Telemonitoring" auf alle Teile der Studie, also sowohl die Messgeräte (Pusten und Uhr) als auch die Benutzung der App inklusive der Fragebögen, des Chats und der Videosprechstunden.

Bei einem Teil der Fragen hast Du die Möglichkeit, zwischen mehreren Abstufungen zu wählen.

Beispiel:

stimme überhaupt nicht zu				stimme voll zu
❑	❑	☒	❑	❑

Bitte lasse keine Antwort aus. Solltest Du Schwierigkeiten haben, eine Frage zu beantworten, dann kreuze diejenige Antwortmöglichkeit an, die am ehesten auf Dich zutrifft. Es gibt keine richtigen oder falschen Antworten.

Bitte blättere nun um und beginne den Fragebogen.

Wenn du an die letzten drei Monate denkst, zu welchem Anteil würdest du sagen, dass du das tägliche Telemonitoring mit App, den Messgeräten und das Ausfüllen der Fragebögen eigenständig bzw. mit Hilfe deiner Eltern durchgeführt hast? Bitte setze auf der Linie hierunter ein Kreuzchen an der Stelle, was am ehesten auf dich zutrifft.

Ich habe das Telemonitoring durchgeführt …

… immer mit Hilfe *… manchmal mit Hilfe, manchmal alleine* *… immer alleine*

0 1 2 3 4 5 6 7 8 9 10

Bitte beantworte die folgenden Aussagen:

	stimme voll zu	stimme eher zu	stimme eher nicht zu	stimme überhaupt nicht zu
Ich bin mit dem Telemonitoring insgesamt gut zurecht gekommen.	❑	❑	❑	❑
Ich konnte die Messungen gut in meinen Alltag einfügen.	❑	❑	❑	❑
Ich wurde durch die Nutzung des Telemonitorings verunsichert.	❑	❑	❑	❑
Ich habe das Gefühl, dass die Behandlung meiner Post-Covid-Erkrankung vom Telemonitoring profitiert hat.	❑	❑	❑	❑
Ich war aufgrund meiner Post-Covid-Erkrankung insgesamt gesundheitlich stark beeinträchtigt.	❑	❑	❑	❑

	regel-mäßig	manch-mal	selten	nie
Mir hat jemand bei der Nutzung des Telemonitorings geholfen.	❑	❑	❑	❑
Ich habe schon vorher Aufzeichnungen zu meiner Gesundheit gemacht (z. B. Herzfrequenz, Kopfschmerztagebuch).	❑	❑	❑	❑
Ich habe mit meiner Kinderärztin/meinem Kinderarzt über meine Messwerte gesprochen.	❑	❑	❑	❑

Seit meiner Teilnahme an der Studie, also in den letzten drei Monaten, hat wegen meiner Post-Covid-Erkrankung Folgendes stattgefunden (Mehrfachauswahl möglich):

	Ja	Nein
Telefonat mit unserer Kinderärztin/Kinderarzt	❑	❑
Praxisbesuch mindestens einmal in unserer Kinderarztpraxis	❑	❑
Hausbesuch unserer Kinderärztin/unseres Kinderarztes bei uns	❑	❑
Anruf beim Rettungsdienst	❑	❑
Vorstellung in einer Notaufnahme	❑	❑
Krankenhausaufenthalt von mindestens zwei Tagen	❑	❑

	Ja	Eher ja	Eher nein	Nein
Würdest Du das Telemonitoring anderen Familien oder befreundeten Kindern/Jugendlichen mit Post-Covid weiterempfehlen?	❑	❑	❑	❑

	Sehr gut	Eher gut	Eher schlecht	Sehr schlecht
Wie beurteilst Du das Telemonitoring insgesamt?	❑	❑	❑	❑
