# Supplementary material for: Feasibility, Adherence, Acceptance and Usability of a Multimodal Telemonitoring for Pediatric Post-COVID Syndrome: A Bicentric Pilot Study
Source: J Med Syst. 2026 May 9;50(1):76. doi: 10.1007/s10916-026-02409-x (PMC13157441; doi:10.1007/s10916-026-02409-x)
Supplement: Supplementary file 6 — Supplementary Material 6 [file 10916_2026_2409_MOESM6_ESM.pdf]

Appendix 4. Custom evaluation questionnaire polling parents' overall experience with telemonitoring.

ID Studien-TN: _______________ Ausfülldatum: _______________

Evaluation coverCHILD Telemonitoring

Fragebogen für Eltern

Sie haben in den letzten drei Monaten gemeinsam mit Ihrem Kind das Telemonitoring im Rahmen der Behandlung der Post-Covid-Erkrankung Ihres Kindes im Kinderzentrum Bethel durchgeführt.

Der Begriff „Telemonitoring" meint im nachfolgenden Fragebogen alle Teile der Studie, also sowohl die Messgeräte (Lungenfunktionsgerät und Smartwatch) als auch die Benutzung der App inklusive der Fragebögen, des Chats und der Videosprechstunden.

Im Folgenden möchten wir durch einige Fragen gerne mehr über Ihre Erfahrungen mit dem Telemonitoring herausfinden. Bei einem Teil der Fragen haben Sie die Möglichkeit, zwischen mehreren Abstufungen zu wählen.

Beispiel:

stimme überhaupt nicht zu				stimme voll zu
❑	❑	☒	❑	❑

Bitte lassen Sie keine Antwort aus. Sollten Sie Schwierigkeiten haben, eine Frage zu beantworten, dann kreuzen Sie diejenige Antwortmöglichkeit an, die am ehesten auf Sie zutrifft. Es gibt keine richtigen oder falschen Antworten.

Bitte blättern Sie nun um und beginnen den Fragebogen.

Wenn Sie an die letzten drei Monate zurückdenken, zu welchem Anteil würden Sie sagen, dass Ihr Kind das gesamte Telemonitoring mit App, den Messgeräten, Videosprechstunden und das Ausfüllen der Fragebögen eigenständig durchgeführt hat? Bitte setzen Sie auf der Linie hierunter ein Kreuzchen an der Stelle, was am ehesten auf Ihre Situation in den vergangenen Monaten zutrifft.

Mein Kind hat das Telemonitoring durchgeführt ...

... immer mit Hilfe	*... manchmal mit Hilfe, manchmal alleine*	*... immer alleine*

0 — 1 — 2 — 3 — 4 — 5 — 6 — 7 — 8 — 9 — 10

Bitte beantworten Sie die folgenden Aussagen:

	stimme voll zu	stimme eher zu	stimme eher nicht zu	stimme überhaupt nicht zu
Wir sind mit dem Telemonitoring gut zurechtgekommen.	❑	❑	❑	❑
Wir konnten die Messungen gut in unseren Alltag integrieren.	❑	❑	❑	❑
Wir wurden durch das Telemonitoring verunsichert.	❑	❑	❑	❑
Ich habe das Gefühl, dass die Behandlung der Post-Covid-Erkrankung meines Kindes vom Telemonitoring profitiert hat.	❑	❑	❑	❑
Mein Kind war aufgrund seiner:ihrer Post-Covid-Erkrankung insgesamt gesundheitlich stark beeinträchtigt.	❑	❑	❑	❑

Bitte beantworten Sie die folgenden weiteren Aussagen. Mit „wir“ sind Sie als gesamte Familie inklusive Ihres Kindes gemeint.

	regel-mäßig	manch-mal	selten	nie
Wir haben unserem Kind bei der Nutzung des Telemonitorings geholfen.	❑	❑	❑	❑
Wir haben schon vorher Aufzeichnungen zur Gesundheit unseres Kindes gemacht (z.B. Herzfrequenz, Kopfschmerztagebuch).	❑	❑	❑	❑
Wir haben mit unserer Kinderärztin/unserem Kinderarzt über die Messwerte meines Kindes gesprochen.	❑	❑	❑	❑

Seit der Teilnahme meines Kindes am Telemonitoring hat wegen seiner:ihrer Post-Covid-Erkrankung Folgendes stattgefunden (Mehrfachauswahl möglich):

	Ja	Nein
Telefonat mit unserer Kinderärztin/Kinderarzt	❑	❑
Praxisbesuch mindestens einmal in unserer Kinderarztpraxis	❑	❑
Hausbesuch unserer Kinderärztin/unseres Kinderarztes bei uns	❑	❑
Anruf beim Rettungsdienst	❑	❑
Vorstellung in einer Notaufnahme	❑	❑
Krankenhausaufenthalt von mindestens zwei Tagen	❑	❑

	Sehr gut	Eher gut	Eher schlecht	Sehr schlecht
Wie beurteilen Sie das Telemonitoring insgesamt?	❑	❑	❑	❑

	Ja	Eher ja	Eher nein	Nein
Würden Sie die Nutzung des Telemonitorings anderen Familien oder Freunden mit Post-Covid-Betroffenen weiterempfehlen?	❑	❑	❑	❑
